# Supplementary material for: Capturing children food exposure using wearable cameras and deep learning
Source: PLOS Digit Health. 2023 Mar 27;2(3):e0000211. doi: 10.1371/journal.pdig.0000211 (PMC10042366; doi:10.1371/journal.pdig.0000211)
Supplement: Attachment — Submitted filename: rebuttal.pdf [file pdig.0000211.s001.pdf]

Response to Reviewers

We would like to thank the reviewers for their valuable feedback, which has helped us improve the paper. Below are our responses to the reviews and how we updated the paper accordingly.

1 Journal Requirements:

1. Please amend your detailed Financial Disclosure statement. This is published with the article. It must therefore be completed in full sentences and contain the exact wording you wish to be published.

   – Done.

2. We ask that a manuscript source file is provided at Revision. Please upload your manuscript file as a .doc, .docx, .rtf or .tex.

   –Done.

3. Please provide separate figure files in .tif or .eps format only and remove any figures embedded in your manuscript file. Please also ensure that all files are under our size limit of 10MB.

   –Done.

4. Figure 2, 3, 4 and 8 include an image of an identifiable person. Please provide written confirmation or release forms, signed by the subject(s) (or their guardian), giving permission to be photographed and to have their images published under a Creative Commons license. You may upload permission forms to your submission file inventory as item type 'Other'. Otherwise, we kindly request that you remove the photograph.

   –We have obtained verbal consent from all the children and their parents who participated in the study about using some of the images captured during the study in publications as long as all the faces are blurred. Since we do not have any written consent, we opted to remove all the images that contain any identifiable person.

5. Please remove any photos of children (Figures 2, 3, 4 and 8) from your submission.

   –Done.

2 Reviewer #1:

- The authors use both MobileNet V1 and V2. What is the reason for that? Have you tried using either one of the other for all the experiments in order to justify why different models are used throughout the paper?

  – For all classification tasks described in the paper, we have trained various state-of-the-art deep learning models such as VGG16, VGG19, in addition to MobileNet V1 and MobileNet V2, employing both transfer learning and training from scratch. Moreover, we have also trained various custom CNN models from scratch. We then performed model selection using the validation sets and the best model for each classification task was used. We only present the test results for the selected models for conciseness. We have clarified this in Section 2, where each classification task is described.

- Why are the models using pre-trained weights from ImageNet if they are being trained from scratch?

  – We trained various models for each classification task, some of which were from scratch and some using transfer learning by loading pre-trained ImageNet weights. However, we only describe the final selected models in the paper. We updated the sentence that mention training from scratch in Section 2 to explain what models were trained and how and which final models were selected using validation.

- Why is a multilabel model needed? To me it seems that a binary classification model could have been used. Moreover, if you train a multilabel system, why is a two-step cascaded system used? A single multilabel model could have been trained including food exposure and food consumption.

  – In the paper, we describe three different classification tasks. The first task is a binary classification task that determines whether an image contains food exposure or not. The second task is a multi-label classification task that determines for a food exposure image, what type of exposure it is. An image may contain multiple food exposures such as an ad and an outlet. The third task was responsible for determining whether an image that depicts food consumption is a self-food consumption by the camera wearer or if it depicts others consuming food, or both. In many food consumption images, the child wearing the camera will be sharing a meal with others and hence an image can belong to both classes "self food consumption" and "others food consumption" at the same time. Finally, we have considered applying a flat classifier that combines the second and third classification tasks, however our empirical results showed that a cascaded approach performs better. This can be attributed to the fact that food consumption doesn't typically overlap with the other classes (outlets and ads) and building a specialized classifier for food consumption would be more successful in differentiating between "self food consumption" and "others food consumption" classes. However, more experiments should be done to verify this in future work.

- The paper has too many lists, which, in my opinion, does not help with the readability of the paper and confuses a bit.

  – We have removed any lists unless we deemed it absolutely necessary to structure the information conveyed.

- Retrieving 2000 images for each query in the food exposure experiment seems to be a lot to me. Have you tried using other configurations for acquiring the images?

  – We did try different settings, however given that the crawling would result in some noisy data, as can be seen in our analysis in Section 2 and Table 1, we settled on 2000 images. Given that this was an automated process and relied on crawling without the need for any manual human labeling, the large number of images did not result in additional burden and allowed us to generate a sufficiently large dataset that can be used to train various deep learning models with various complexities before choosing the best model to employ.

- The authors mention a few times in the paper that classes should be balanced and that some images are removed in order to have this aforementioned balance in the datasets. Why is this needed? If you're using TensorFlow you could use the class_weight parameter to benefit from more images while not having balanced classes.

  – We opted to work with balanced datasets to avoid biased models. Given that all our datasets were large in size, we could afford to perform undersampling, which is a common technique to deal with imbalanced data. However, other techniques can be also employed as the reviewer suggests such as oversampling as well as instance weighting.

- There is too much detail given for some specific things that I would recommend removing. Particularly, those lines in which the evolution of MobileNet from V1 to V2 is explained, as well as those lines related to how much disk space is used when installing libraries or with the datasets used.

  – We removed all unnecessary details as kindly suggested by the reviewer,

- What would happen in the case that these models are used in a completely different country in terms of food culture? The models seems to be trained on a specific local food variety. I wonder how this would work on countries like Japan, USA, Mexico, etc. Wouldn't it be better to train the model on a dataset with a higher heterogeneity?

  – We believe that our models should be able to generalize well to other countries or food contexts. While our first model relied on crawling images of food items related to Lebanon and Tunisia, which are the setting of our user studies, given that these were Web images and given that international cuisines are very popular in these countries, and that the

ingredients in food items whether local or international have a lot in common, we believe the model should generalize well to other countries. In addition, images of outlets and advertisements should not differ much from one country to another, except perhaps for text language. We have made all the data we collected and our models publicly available so other researchers can also validate this. All other models in this paper were trained using a mixture of publicly available datasets and our own collected data and thus should also be heterogeneous enough to generalize to other settings or contexts.

3 Reviewer #2:

- Line 72: Missing "(a)" for food consumption image in Figure 2

  – Thanks for pointing this out. It's fixed in the paper now.

- Line 226: "desktop application extracts a frame every 10 seconds" What is the rationale behind this time frame, do similar tools follow this frame? It would be useful to clarify this for the reader.

  – The rationale behind extracting a frame every 10 seconds is to avoid too many duplicate images capturing the same scene. Given the setting of our studies which involve school children capturing footage through wearable cameras, we believe a window of 10 seconds would strike a good balance between eliminating unnecessary duplicates and at the same time making sure we capture all distinct exposures. Our post analysis of the data collected in our user study in Tunisia confirms this intuition. We have added this rationale in the paper in Section 2.1.2.

- Great example of user-centered research to inform best practices in developing a tool that is culturally appropriate and acceptable in low-middle income countries like Lebanon and Tunisia. This is a timely study given the explosive increase in obesity figures in the region. Further validation of this tool among other age groups (adolescents and adults) could pave the way for a shift in dietary intake methodology. The existing gold standard for measuring dietary intake data is weighed food records and in some instances 24 hour recalls are used to validate novel approaches such as digital cameras. Are there plans to validate the measurement tool using other dietary assessment methods? Authors mentioned future plans to classify healthy food items using NOVA. It may be worth considering to incorporate volume estimation, and nutritional analysis to further strengthen this as a potential tool to replace conventional dietary assessment methods.

  – We thank the reviewer for the valuable suggestion. In order for us to validate the camera component of the study, the 24 hour recall should cover the same period in which the camera was used to capture food exposure. While we did do recall-based assessment, the time period covered

was different from that where the cameras were used. However, we will attempt this in our next user study that takes place in Lebanon.

4 Reviewer #3:

- I know that the term "AI" has been used elsewhere, but I find it silly and unscientific. Nothing about this model represent 'intelligence'. "Machine learning" is the more accepted term in academia.

  – We thank the reviewer for pointing this out. We have replaced the term AI with machine learning everywhere as suggested.

- In general I see no problem with training a model using images scraped from the web (there is a slightly thorny issue of copyright that I hope that authors considered, but I don't think it is a scientific issue). However, I am a bit concerned that the accuracy reported throughout the paper is that of the test set derived from the web images. That is not a measure of accuracy that is relevant to the deployment of the system. We have used and coded wearable camera data, and the images tend to be slightly blurry, not have the full item of interest in the frame, have substantial variance in lighting, etc. You have a dataset that was used for training the classification model – why was this not used as the test data for the detection model? And, if it wasn't, you could compute and report accuracy post hoc?

  – In the paper, we describe three different machine learning models. The first, which aims to classify whether an image contains food exposure or not, used crawled Web images for training while the other two relied on a mix of publicly available data and our own data that was manually labeled using crowdsourcing. Our study protocol dictates that we deploy the first machine learning model before we collect data. The model was used to filter out any non-food related images that we collect through the wearable cameras. The retained images were then screened by the participants' parents manually to remove any other images they did not wish to keep. Only then we were given access to any images, which means that the images we finally collected all contain food exposure. Given these constraints and the lack of publicly available data, we had no choice but rely on Web images as means to generate training data for our first machine learning model.

  The above comment also applies to the classification models. The only metric that matters is if the model is accurate in the type of data you want to deploy it to (i.e., free-living wearable camera data). Therefore, only these images should be contained in the test set. I know that this is somewhat tested in a small case study, but it should also have been part of the model evaluation.

  – For the other two classification methods, the test data was constructed

from data collected through wearable cameras and that were manually labeled through crowdsourcing. In addition, as the reviewer pointed out, we evaluated the end-to-end system in a small case study in Tunisia. We plan to do further user studies in other locations and to conduct various analyses to evaluate our system and validate the findings through extrapolation with other data collected as part of the study.
